# Photodynamic Therapy as an Adjunct to Resective and Regenerative Surgical Treatment of Peri-Implantitis: A Prospective Cohort of 72 Patients Followed for 18 Months

**DOI:** 10.3290/j.ohpd.c_2078

**Published:** 2025-06-03

**Authors:** Volkan Arısan, Alper Sağlanmak, Ata Anıl, / S. Volkan Arıcı, / Anton Sculean

**Affiliations:** a Volkan Arısan† Professor, Faculty of Dentistry, Department of Oral Implantology, University of İstanbul, İstanbul, Türkiye. Principal investigator, performed part of the experiments, collected data, performed statistical analyses, prepared and reviewed the manuscript.; b Alper Sağlanmak† Assistant Professor, Faculty of Dentistry, Department of Oral Implantology, University of İstanbul, İstanbul, Türkiye. Principal investigator, concept development, study and manuscript design, manuscript preparation, edited and reviewed the manuscript.; c Ata Anıl Professor, DTMD University, Wiltz, Luxembourg. Performed part of the experiments, edited and reviewed the manuscript, contributed substantially to discussion.; d S. Volkan Arıcı Doctor of Oral Implantology, Private Practice, İstanbul, Türkiye. Performed part of the experiments, edited and reviewed the manuscript.; e Anton Sculean Professor, Department of Periodontology, University of Bern, Bern, Switzerland. Rdited and reviewed the manuscript contributed substantially to discussion. †Joint first authors.

**Keywords:** dental implants, guided bone regeneration, implantoplasty, peri-implantitis, photodynamic therapy.

## Abstract

**Purpose:**

To evaluate the efficacy of photodynamic therapy (PDT) as an adjunct to resective and regenerative surgical peri-implantitis treatment (open flap debridement via scaling and smoothening of the implant surface [implantoplasty]) combined with guided bone regeneration (GBR) in a patient cohort of a university clinic.

**Materials and Methods:**

Seventy-two patients were treated with either conventional therapy (CON) or conventional therapy plus PDT. CON included mechanical debridement, implantoplasty, and GBR. Clinical parameters, including marginal bone level (MBL), probing pocket depth (PPD) and bleeding on probing (BOP) were assessed at baseline, 6, 12, and 18 months. The primary outcome was the resolution of the infection using a composite success criterion.

**Results:**

After 18 months, infection resolution rates were 75% for CON and 80% for PDT groups (p = 0.75). Kaplan-Meier survival analysis showed no statistically significant difference for the infection resolution between groups (log-rank test, p = 0.6221). Both groups demonstrated statistically significant MBL gain after 6 months (mean 2.59 mm ± 1.25), with no statistically significant differences between groups throughout the follow-up. PPD was statistically significantly lower in the PDT group (two-way ANOVA, p = 0.018). BOP scores decreased initially but showed an increasing trend in both groups over time (chi-squared test, p = 0.045), with no statistically significant differences between groups.

**Conclusion:**

PDT as an adjunct to conventional peri-implantitis treatment with GBR resulted in statistically significantly lower PPD values. However, no additional benefits were observed for infection resolution, maintenance of infection-free status, MBL or BOP. Initial improvements in both groups followed by gradual recurrences in clinical parameters over 18 months.

The increase in the human life expectancy has led to a dramatic increase in the number of dental implants placed for the rehabilitation of all types of tooth loss. Despite the high success rates of the osseointegration, dental implants have been reported to demonstrate inflammatory signs and relevant tissue destruction – called “peri-implantitis” akin to “periodontitis” – with a prevalence rate up to 57%.^8,15,41,42,45,50,51,55^ In a systematic review and meta-analysis, the weighted mean prevalence of peri-implantitis was estimated to be around 22% of implants and 12% of patients.^18^

This inflammatory state poses a risk for the longevity of the implant and the overall well-being of the individual, since the lesions are usually chronic and progress faster than do periodontal lesions.^25,39^ The treatment of peri-implantitis is challenging due to the complex implant surface topographies, which include randomly created lobular spaces that can potentially accumulate dental plaque once exposed to the oral environment.^28^ Unlike the conventional methods of periodontal treatment, i.e., mechanical debridement followed by disinfection via a suitable medium, the biofilm on the rough surfaces of the implant and the surrounding area is difficult to remove. This has been suspected to be the major cause in the high recurrence rates of periimplantitis.^16^

Traditional treatment approaches, such as mechanical debridement and antimicrobial agents, show suboptimal results in achieving long-term success.^53,54^ Conventional mechanical methods are easy to perform but unable to remove bacterial by-products in and outside of the infected region.^57^ Elimination of the infectious elements, i.e., biofilm, bacteria, viruses and fungi, reduces the probing pocket depth (PPD) around the infected implant, impeding bacterial adhesion and reinfection.^31^ However, it has not been possible to achieve this by mechanical debridement alone.^53^ Chemical methods have been shown to be effective in-vitro, but their efficacy was questioned under challenging clinical conditions that compromise their reach to the infected areas of the disease.^59,61^ Laser irradiation deploys controlled energy to the target area, and conversion of the energy to heat provides selective thermolysis of the pathogens.^3,20^ Unfortunately, the reach of the beam is hampered by the threads of the dental implant, especially when the implant is sub-optimally positioned.^28^

Photodynamic therapy (PDT) has been used to decontaminate the implant surface and the infected surrounding area.^47^ PDT employs a photosensitive agent activated by a specific wavelength of a laser light, so that the resulting reactive oxygen particles disrupt biofilm, destroy the bacterial cell wall, and detoxify the implant surface as well as the surrounding area.^11,12,17^ The method has shown promising results in different fields of medicine.^19^ However, conflicting results have been obtained in the treatment of peri-implantitis,^6^ especially when combined with a reconstructive approach including guided bone regeneration (GBR).

Consensus is still lacking on the optimal protocol for reconstructive surgery to treat peri-implantitis, with no single method proving to be universally superior. The aim of this study was to compare the efficacy of PDT as an adjunct to resective and reconstructive surgical treatment of peri-implantitis including mechanical and chemical debridement, implantoplasty, and GBR.

## MATERIALS AND METHODS

The study protocol was approved by the ethics committee of the Faculty of Dentistry of the Istanbul University (Protocol no: 735/357-80) and was conducted in accordance with the ethical principles outlined in Declaration of Helsinki for studies involving human subjects as revised in 2013. STROBE guidelines were followed in the preparation of this manuscript.

### Sample Size Calculation

The primary outcome of the study was the resolution of the infection (absence of bleeding on PPDs of 5 mm or less, and no statistically significant radiographic bone loss). To test the null hypothesis that the adjunctive use of photodynamic therapy (PDT) in the treatment of peri-implantitis does not result in a statistically significant difference in infection resolution compared to conventional treatment (reconstructive treatment of peri-implantitis including mechanical and chemical debridement, as well as GBR), data from a previous study were utilised for the calculations, yielding an effect size of 0.344. Using dedicated software (G-Power version 3.1; Düsseldorf, Germany) a total sample size of 68 was determined to achieve the critical χ^2^ of 3.84 and a λ of 7.86 with a statistical power of 0.80.

### Patient Selection Criteria

All patients from the Department of Oral Implantology, Faculty of Dentistry, Istanbul University, Türkiye who had been previously treated with dental implants at the same clinic were investigated between May 2018 and November 2022. Those with an osseointegrated titanium implant supporting a functional crown with a function time of ≥ 12 months and complained of pain, swelling, and pus at the implant region were considered for the study when a radiographic bone loss of ≥ 3 mm was visible on the patient’s panoramic radiograph. The patients were clinically examined by the calibrated examiner (A.S., κ = 0.82) using a plastic periodontal probe. The diagnostic criteria for peri-implantitis were based on an expert consensus^13^ and included: presence of suppuration from the peri-implant sulci or ≥ 5 mm probing depth, or presence of bleeding on probing (BOP) and accompanied by a radiographic peri-implant MBL of ≥ 3mm visible on the patient’s panoramic radiograph. Commercial software (Romexis, Planmeca; Helsinki, Finland) was used for measuring MBL. Based on an established method,^29^ the software was calibrated considering the known implant length and distance from visible bone contact on the implant body; the implant platforms were measured from the distal and the mesial aspect. The arithmetic mean was recorded as the MBL of the implant.

All patients were informed about the prognosis and treatment options of the disease, and patients accepting surgical treatment of peri-implantitis were included following written consent regarding the procedures to be undertaken.

### Exclusion Criteria

The criteria for exclusion were age < 18 years, consent not given, uncontrolled diabetes mellitus (HbA1C level ≥ 6.4), active oncological treatment, heavy smokers (smoking ≥ 10 cigarettes/day), other chronic diseases that may interfere with bone healing, dental implants other than standard-sized titanium screw-type with a rough surface (mini [diameter < 3.3 mm], extra short [length < 7 mm], and/or wide [diameter > 5.5 mm], zirconia implants and machined surface implants), and untreatable implants (severe malposition, a baseline peri-implant MBL of more than half of the implant length, inconvenient access to the implant site, absence of keratinized mucosa around the implant or mobile mucosa surrounding the implant or other circumstances impeding GBR surgery around the affected implant).

### Statistical Analysis

The primary outcome was infection resolution (absence of bleeding in PPDs of 5 mm or less, and no significant radiographic bone loss) and secondary outcomes were the MBL, PPD and BOP changes. Descriptive statistics including mean, standard deviation (SD), range and 95% confidence intervals were calculated. Normality of the data distribution was assessed using the Kolmogorov-Smirnov test. The similarity of the baseline group characteristics was investigated using the t-test, Mann-Whitney U-test and chi-squared test. Kaplan-Meier survival curves with the log-rank (Mantel-Cox) test were used for the assessment of the treatment outcomes “infection resolved” or “infection not resolved” as well as for patients who were lost to follow-up. The effect of the treatment methods (CON and PDT) on the interval values (MBL and PPD) over time was investigated by general linear mixed models (two-way ANOVA). All analyses were performed using GraphPad Prism software (version 9 for Mac, GraphPad Software: San Diego, CA, USA), and p < 0.05 was considered the threshold of statistical significance.

### Surgical Intervention

Surgical treatment of peri-implantitis was based on the recommendations by an expert consensus.^25^ To suppress infection, all patients were administered systemic antibiotics (amoxicillin 1000 mg) for of 5 days starting two days before surgery.

A full-thickness flap extending to 1–2 teeth distally and mesially to the infected implant was raised. A releasing incision following the horizontal base of the raised flap was made at this stage to prevent excessive post-operative hematoma and sustain a passive flap closure at the end of the surgery. The surface of the implant was thoroughly cleaned of debris via plastic scalers and any granulation tissue was removed by curettes. The procedure known as “implantoplasty” was accomplished by flattening and smoothening the exposed implant body threads via the diamond and Arkansas-stone burs.^60^ The surgical site and the exposed implant surface were ensured to be free from any debris or infective material. The site was copiously irrigated with saline (NaCl), and a piece of gauze soaked with hydrogen peroxide was nestled onto the infected area for a duration of 1 min.

Photodynamic therapy (PDT) was applied in patients who had provided prior consent. The procedure followed the written protocol of a commercial integrated system for dental implants (Helbo, Bredent; Senden, Germany). The photosensitizer (Hello Blue, Bredent) was applied to and around the infected area, including the implant surface. Care was taken to create a thin layer without bubbles, and a minimum of 1 min was allowed to ensure the effective coating. The area was irradiated using the tip of the pen-shaped diode laser (Helbo Theralite, wavelength: 380–700 nm, Bredent) in a circular manner for a duration of 1 min.

The area was copiously irrigated with saline solution, the exposed defect space was grafted via a particulate xenograft (Bio-Oss, Geistlich Pharma; Wolhusen, Switzerland), and a resorbable collagen membrane (Biogide, Geistlich Pharma) was applied onto the grafted defect area to maintain the conditions for guided bone regeneration. Primary flap closure was achieved by intermittent non-permeable stitches. In 36 implants (46.8%; 26 CON and 10 PDT), it was possible to remove the prosthesis. All implants underwent non-submerged healing via a healing abutment, existing prosthesis or final abutments that could not be removed. The screw access hole was filled with a filling material to prevent plaque accumulation.

Post-operative analgesia was provided by NSAIDs and cold application onto the side of the face with the surgical site to reduce swelling. Patients were instructed to maintain a strict oral hygiene routine except for the surgical site. Chlorhexidine mouthrinse was administered daily to disinfect of the surgical site. The sutures were removed after 10 days, at which point chlorhexidine use was discontinued, and patients resumed their regular oral hygiene practices.

All prostheses were evaluated for cleanability and hygiene access, based on the patient’s daily routines. Bridges with overlapping contours were flattened and shaped to ensure easy access all around the implant via floss and/or interdental brush. Prostheses were re-screwed or cemented after 32 (IQR:11) days (median) following the removal of the sutures. Following the surgical intervention, patients were scheduled for follow-up examinations at 6-month intervals. All patients were recalled at 6-month intervals and a new panoramic radiograph was taken at each visit for the evaluation of MBL according to an established method.^5,29,56^ Intraoral examination and periimplant area inspection were done, including PPD and BOP, and digital examination via index fingers was performed to determine the presence of any pus around the area. Throughout the study, radiographs were obtained using the same device (Instrumentarium, Orthopantomograph OP30; Tuusula, Finland) and dosage (72 kVp, 8 mA, and an exposure time of 5 s).

**Fig 1:**
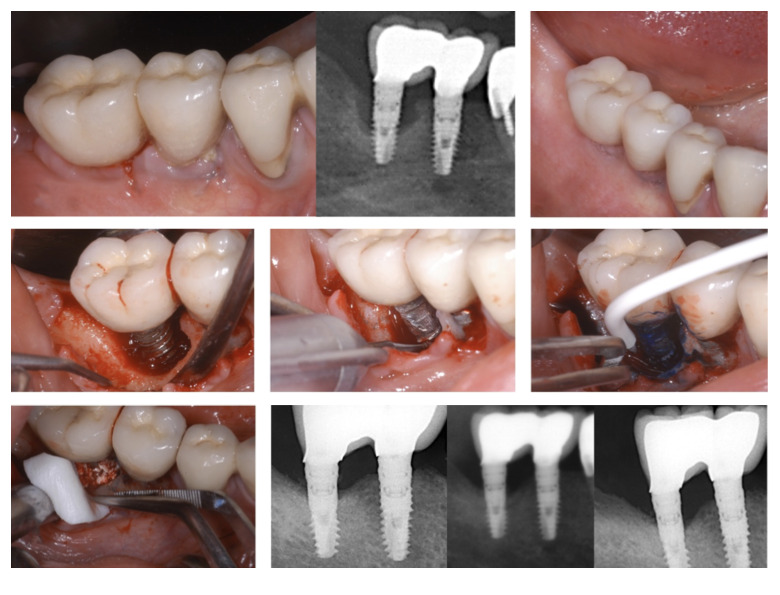
Treatment sequence of an implant in the region #46. Clinical and radiographic measures indicated peri-implantitis. (a) Clinical view after the initial treatment. (b) Mechanical debridement of the granulation tissues, calculus (c) and implantoplasty (d). PDT application (only for the implants assigned to the PDT group) (e). Guided bone regeneration (f). Marginal bone level changes measured at the 6-, 12-, and 18-month (from left to right) examinations.

The presence of any progressive marginal bone loss was evaluated visually using the subsequent panoramic radiographs displayed on computer monitor (Fig 1).

## RESULTS

A total of 144 implants in 144 patients were diagnosed with peri-implantitis; 118 patients gave their consent for treatment. During the oral examination, 6 implants in 6 patients were found to be mobile and thus removed. The remaining 112 patient included of 68 females and 44 males (median age: 45 [IQR: 54] years). Mean time in function of the implants was 27.4 (± 8.3) months.

Initial treatment, consisting of plaque and tartar removal using ultrasonic and plastic scalers combined with disinfection with chlorhexidine and hygiene motivation, was performed in 112 patients. Adjustments to the implant prostheses were made to improve cleanability, and patients were instructed in the use of dental hygiene tools, such as floss, water jets, and mouthwash. All patients were scheduled for a 3-month recall.

No patients complained of an urgent or unexpected event. However, due to loosening of their cement-retained implant prostheses, 21 patients requested re-cementation following the period of initial treatment. Twenty patients dropped out, while the remaining 92 returned after a median healing period of 3 months and 19 days (IQR: 21). Six implants did not exhibit any signs of infection, were considered successfully treated, and were excluded from the study. Resolution of the infection was not achieved in 82 patients; 10 of their implants were deemed untreatable due to conditions such as severe swelling, pain, pus and/or advanced bone loss. These implants were removed using reverse-torque or trephine burs. The remaining 72 patients (n = 72 implants) proceeded to surgical intervention. Among them, 22 patients consented to adjunctive photodynamic therapy (PDT). The recruitment of the patients for the study proceeded beyond the initially determined sample size, and included additional control (CON) treatments, in order to increase the statistical power of the study.^35^ Thirty-nine implants were classified as standard diameter (3.75, 3.8 mm), 12 as wide diameter (4.5, 4.8. and 5.5 mm), and 21 as narrow diameter (3.2–3.5 mm), with lengths ranging from 8 to 14 mm. The locations of the implants according to the groups are shown in Table 1.

**Table 1:** Distribution of the implants by jaw and region, with relation to the treatment cohorts

	Anterior region (canine-to-canine)	Posterior region (premolars and molars)
Mandible	17 (13 CON and 4 PDT)	18 (13 CON and 5 PDT)
Maxilla	14 (8 CON and 6 PDT)	23 (16 CON and 7 PDT)


Fifty-two implants were single standing units and 20 were serving as the anchor in a two-unit bridge. A total of 41 implants provided support for cement-retained prostheses, while 32 implants supported screw-retained prostheses. The prostheses were predominantly metal-ceramic (59) or zirconia (13). The manufacturers of the implants are given in Table 2.

**Table 2:** Commercial manufacturers of the implants in the cohorts

Manufacturer*	CON	PDT
Southern	10	
Biohorizon	9	3
Frialit II Friadent	6	8
Straumann BL	7	
Nobel	7	4
Xive, Dentsply	5	7
Nucleoss	4	
Thommen Medical	2	
**Total**	50	22
*Southern: Irene, Republic of South Africa; Biohorizon: Birmingham, AL, USA; Straumann BL: Straumann BL, Basel, Switzerland; Firalit II Friadent and Xive: Dentsply Sirona, Charlotte, NC, USA; Nucleoss: İzmir, Turkey; Thommen Medical: Grenchen, Switzerland.

After 6 months, a total of 2 patients were lost to follow-up, leaving 70 patients for evaluation. At the 6-month mark, both treatment methods demonstrated high success rates in resolving infection (97.9% for the CON group and 95.5% for the PDT group). However, these success rates declined over subsequent follow-up intervals, reaching 76.6% for the CON group and 95.23% for the PDT group (p = 0.08). After 18 months of follow-up, the final infection resolution rates were 75% for the CON group, and 80% for the PDT group, with no statistically significant difference between the two groups (p = 0.75; Table 3).

**Table 3:** Resolution of the infection % in the control and PDT groups

		CON	PDT	p
Number of implants at risk during the investigation period		48	22	
6 months	Infection resolved	47 (97.91%)	21 (95.45%)	0.53
	Infection not resolved	1 (2.09%)	1 (4.55%)	
12 months		47	21	
	Infection resolved	36 (76.59%)	20 (95.23%)	0.08
	Infection not resolved	11 (23.41%)	1 (4.77%)	
18 months		36	20	
	Infection resolved	27 (75%)	16 (80%)	0.75
	Infection not resolved	9 (25%)	4 (20%)	
Number in brackets represent cumulative %. Fisher’s exact test for resolution of infection.

**Fig 2:**
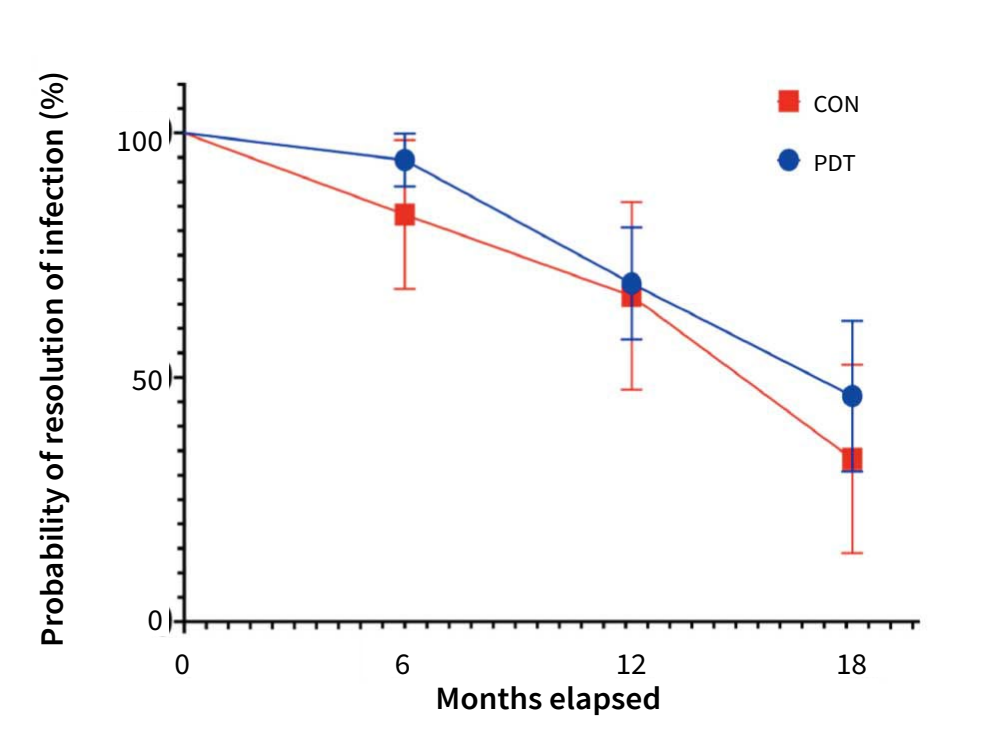
Kaplan-Meier survival curves (event = infection not resolved) of peri-implantitis treatment with (PDT) and without adjunct PDT treatment (CON). No statistically significant differences were detected between the CON and PDT groups (log-rank (Mantel-Cox) test, p = 0.6221, chi-squared = 0.2430).

PDT demonstrated a slightly higher probability of infection resolution but the differences between the Kaplan-Meier survival curves of PDT and CON (event: “infection not resolved”) was not statistically significant (log-rank/Mantel-Cox test, p = 0.6221, chi-squared=0.2430) (Fig 2).

**Fig 3:**
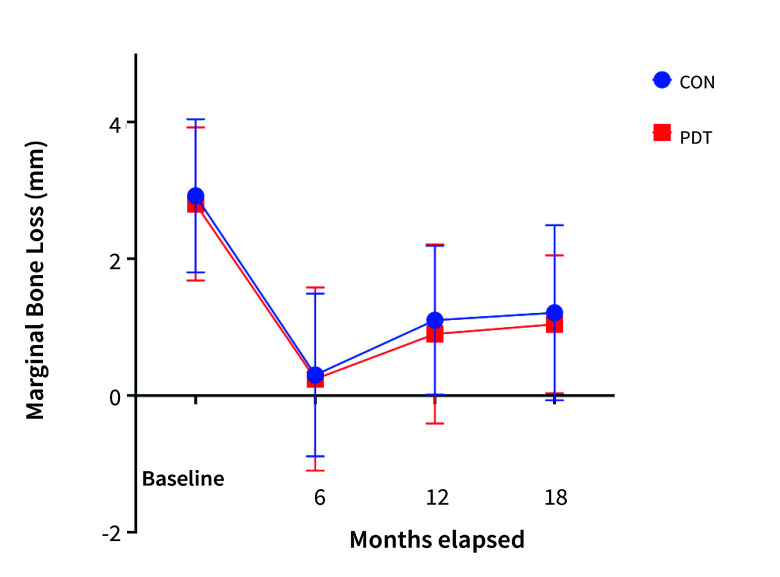
Measured MBL changes in the CON and PDT groups. The changes over time were statistically significant (p < 0.0001), but the differences were not statistically significant between the CON and PDT groups.

Initially, the mean marginal bone loss (MBL) was 2.92 mm (± 1.09) in the CON group and 2.8 mm (± 1.11) in the PDT group, with no statistically significant difference between the groups (Mann-Whitney U-test, p = 0.26). The guided bone regeneration procedures resulted in an average bone gain of 2.59 mm (±1.25) after a 6-month healing period, which was also similar between the groups (Mann-Whitney U-test, p = 0.28). However, MBL did not appear to stabilise during subsequent follow-up periods. The change in MBL over time was statistically significant (two-way ANOVA, F[3, 245]=52.38, p < 0.0001). Neverthelss, no statistically significant differences were observed between the groups at any follow-up (Table 4, Fig 3).

**Table 4:** Mean MBL (mm) in the groups throughout the follow-ups

	CON	PDT
Mean	SD	N	Mean	SD	N
Baseline	2.92	1.12	50	2.8	1.12	22
6 months	0.3	1.19	48	0.24	1.34	21
12 months	1.1	1.09	47	0.9	1.31	21
18 months	1.21	1.28	36	1.04	1.01	20


**Fig 4:**
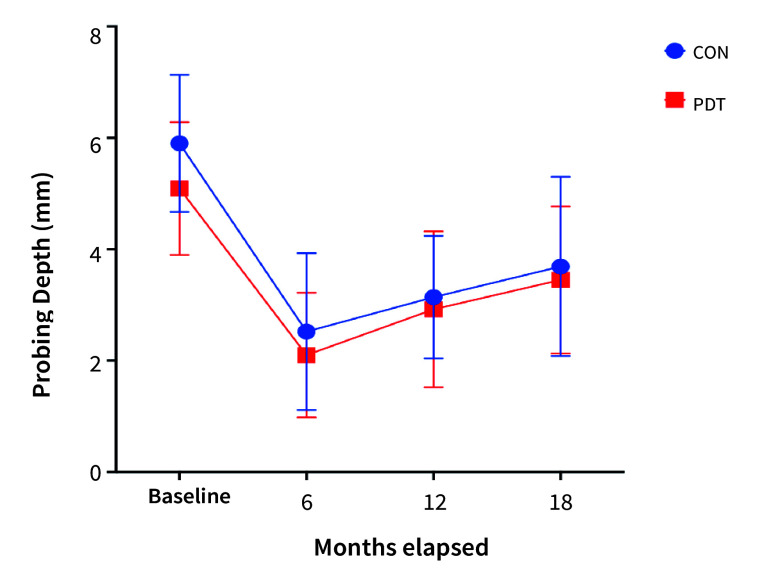
PPD measurements in the CON and PDT groups. The changes in PPD over time were statistically significant (p < 0.0001); the PDT group revealed statistically significantly lower PPD (p = 0.018).

The mean PPD at the beginning of the study was similar across groups, measuring 5.9 mm (±1.23) in the CON group and 5.09 mm (±1.19) in the PDT group (Mann-Whitney U-test, p = 0.45). While a reduction in PPD was observed after 6 months, it slowly increased during subsequent follow-ups (Table 5). The changes in PPD over time were statistically significant (two-way ANOVA f[3,245] = 65.77, p < 0.0001) and the PDT group consistently showed statistically significantly lower PPD values compared to the CON group (two-way ANOVA, F[1, 245] = 5.677, p = 0.018) (Fig 4).

**Table 5:** PPD measurements in the CON and PDT groups

	CON	PDT
Mean	SD	N	Mean	SD	N
Baseline	5.9	1.23	50	5.09	1.19	22
6	2.52	1.41	47	2.1	1.12	21
12	3.14	1.1	47	2.92	1.4	21
18	3.69	1.61	36	3.45	1.32	20


Similarly, the bleeding on probing BOP scores, which were remarkably high at the beginning, showed a statistically significant reduction after 6 months but then exhibited an increasing trend in both groups during subsequent follow-ups. The change in BOP over time was statistically significant (Friedman test; p = 0.045). However, no statistically significant differences were observed between the two groups (Table 6).

**Table 6:** BOP scores (+/-) in CON and PDT groups

	CON	PDT
Positive sites	N	Positive sites	N
Baseline	41 (82%)	50	15 (68.18%)	22
6 months	11 (22.91%)	47	5 (22.72%)	21
12 months	21(51.21%)	47	9 (42.85%)	21
18 months	21 (65.62%)	36	9 (52.94%)	20


## DISCUSSION

The efficacy of photodynamic therapy (PDT) as an adjunct to conventional peri-implantitis treatment combined with guided bone regeneration (GBR) was investigated in this cohort of 72 patients. A comprehensive set of peri-implant health parameters was evaluated for over an 18-month period, allowing robust comparisons despite patient drop-outs. Both groups demonstrated high treatment success at the 6-month mark; however, these outcomes were not sustained over the subsequent 12 months. Notably, with the exception of PPD, the evaluated parameters showed no significant differences between the groups across all time points.

Implant surface decontamination in the treatment of peri-implantitis remains a complex challenge due to the formation of biofilm on the infected area. This biofilm is primarily composed of bacteria, fungi, extracellular polysaccharides, proteins secreted by microorganisms and host tissues, and metals leaching from the implant.^46^ Insufficiency of the current methods was presumed as the cause of recurrences and the reported poor outcomes of peri-implantitis treatment.^14,58^ In PDT, the activated photosensitising agent produces reactive oxygen species that selectively eradicate bacteria and by products on the exposed implant surface and the infected area.^27^ In this study, PDT was applied as an adjunct following the removal of the exposed rough implant surface via implantoplasty,^9,40^ and therefore the risks of implant surface decontamination can be considered eliminated.^36^ Recurrence of the infection following peri-implantitis treatment has been frequently reported and incomplete decontamination was reported as the suspect.^25,28,31,59^ The absence of any such acute reactions within the 18 months of follow-up may reflect the decontamination efficacy of PDT in this study.

While there was no difference between the groups in MBL and BOP parameters, PPD was found to be statistically significantly lower in the PDT group. A similar outcome was reported by another clinical study with 22 patients in whom PDT was adjunctively applied for treatment of periimplant mucositis.^2^ A reduction of ~5mm in PPD was reported after 9 months of healing, which was attributed to the strong bactericidal effect of the PDT. Another clinical trial confirmed the reduction of PPD (3.9 ± 1.2 mm) following PDT as an adjunct to open flap debridement, but the results were statistically significant, probably due to the heterogeneity of the study population (n = 24), which included heavy smokers and diabetics.^1^ A reduction of PPD as a result peri-implantitis treatement may have positive consequences in the long term, since the pathogenic anaerobic bacteria are less likely to reside in the shallow periimplant pockets.^63^ Taken together with the supporting findings of a meta-analysis,^21^ it can be concluded that adjunct PDT application may provide a greater reduction of PPD than conventional debridement alone.

BOP, a long-recognised indicator for assessing tissue health around teeth^34^ and implants,^33^ decreased after treatment, but was found to increase over the subsquent examination intervals. A similar outcome was obtained by a clinical trial with a sample of 40 peri-implantitis patients, in whom implants treated by adjunct PDT demonstrated a reduction of BOP (60%) with statistically significant differences after 24 months, as compared to the conventional treatment (30%). When compared to a topical chlorhexidine gel application, PDT provided statistically significant reduction of BOP in a sample of 40 patients with 52 peri-implantitis sites in the 3-month follow-up period of another clinical trial which primary focused on changes in the microbiota.^48^

Microbial sampling may not be as important as once assumed for the longitudinal assessment of periodontitis and peri-implantitis treatment.^30,37^ The bacterial spectrum and load may differ over time even in the same individual and site, making relevant conclusions only temporarily valid, since the presence or absence of some pathogens was not found to be correlated with the clinical improvements.^4,30,37^ Therefore, such analyses were not undertaken in this study; the focus was instead on clinical (PPD and BOP) and radiographic parameters (MBL). Nevertheless, the comparison of MBL as a criterion of success in the regenerative treatment of peri-implantitis defects is also complicated due to differences in the steps and materials utilised in previous studies.^43^ While a significant gain of MBL was achieved by various grafts, predictability of the results seemed to be provided by coverage with a membrane, i.e., GBR.^44,50^ GBR is recommended for contained bone defects (with no fewer than 3 walls) around the implants following proper surface decontamination;^43^ that study achieved these goals by conventional debridement, implantoplasty and GBR to restore the damaged peri-implant bone. Hence, procedures such as implantoplasty and apically positioned flap may result in food-trap gaps or unacceptable esthetics, requiring augmentation.^43,64^ The comparison of the success of GBR was based on the quantification of the radiographic bone gain and expressed as MBL.^43^ A study which employed reconstructive therapy in combination with implantoplasty achieved an 86% success rate, and 2.64 ± 1.59 mm radiographic bone fill was observed at the end of a 1-year follow-up period.^23^ The study by Lin et al^36^ found that implantoplasty and regenerative therapy did not make a statistically significant difference in clinical parameters but resulted in more radiographic bone gain (3.08 mm). In this study, GBR statistically significantly increased the bone levels around the implants in both groups (mean: ~2.6 mm) but this was not maintained during the consecutive intervals (mean loss ~0.9 after one year) in either group. A comparable outcome was reported by a similar study,^26^ suggesting remodeling and/or re-infection of the site as a cause. Moreover, it is important that MBL assessment be based on a comprehensive and standardised approach, since methods used for the measurement of MBL are futher complicated by the addition of a grafting material around the affected implants.

Instead, infection resolution was proposed as a composite success criterion for the comparison of various treatment methods.^24^ Many studies have reported disease resolution using different definitions. Some studies considered the resolution of peri-implant mucosal inflammation (no areas with BOP) as a success criterion,^7,10,32^ while others only considered the absence of areas with PPD ≥ 6 mm.^38^ Others have defined resolution considering different PPD thresholds as clinical endpoints, with no BOP and no further bone loss.^10,62^ Renvert et al^52^ considered the absence of any detectable MBL, BOP, suppuration and a PPD of ≤ 5 mm as the criteria of success, similar to that of this cohort. To the extent of the authors’ knowledge, the recent study is the first to apply such criteria for the steps undertaken which included PDT for the treatment of peri-implantitis. In this study, both groups showed high success rate of > 95% at 6 months, but signs of infection were observed in the consecutive examination intervals. As a result, the probability of survival without infection dropped to an average of 50% after 18 months. Similar outcomes were reported by previous studies employing various methods and techniques, and were related to factors such as inefficacy of the decontamination and, if applied, regenerative procedures, supportive maintenance therapy, oral hygiene habits, and the host response.^22,38,49,62^ A direct comparison of the present findings with others is challenging due to the differences of the procedures and measures used for success assessment. However, the 18-month results of this cohort are conclusive that the procedures undertaken are partly effective for the elimination of infection. The mechanisms of healing and re-infection following peri-implantitis treatment should be elucidated through further research.

This study has several limitations. The lack of randomisation may have introduced bias in the use of PDT and thus the relevant results. Also, the repeatability and validity of the results may not be ensured in all peri-implantitis cases, due to the strict inclusion and exclusion criteria employed in this study. In PDT, different manufacturers of the device used, different methodologies employed with other settings and armamentarium, may produce different results. The potential impact of gingival recessions and the width of keratinized attached mucosa on the prognosis was not investigated, which could have provided deeper insights into treatment outcomes. Also, the current study was authorised as a parallel cohort investigation, and systematic antibiotics were administered in line with the standard protocols for managing peri-implantitis.^8^ Concurrent use of systemic antibiotics limits the ability to isolate the specific effect of PDT on the resolution of inflammation, so that randomised controlled clinical trials are needed to evaluate PDT without adjunctive systemic antibiotics to better understand its independent contribution to peri-implantitis treatment outcomes.

## CONCLUSION

For the treatment of peri-implantitis, PDT as an adjunct to conventional mechanical debridement, implantoplasty, and GBR, yields lower PPD. However, no additional benefit of PDT was observed for infection resolution and maintenance of the infection-free status, probability of re-infection, MBL, and BOP. In both groups, the initial improvements were followed by gradual recurrences in clinical parameters over 18 months.

## ACKNOWLEDGEMENTS

The authors thank Dr. Nuriye Ertan Açıkgöz and Dr. Fahri Açıkgöz for their contribution to the statistical analyses conducted in this study. This study was partly supported by the Istanbul University Research Fund (ID: 34832).
